# DNA Methylation Mediates the Discriminatory Power of Associative Long-Term Memory in Honeybees

**DOI:** 10.1371/journal.pone.0039349

**Published:** 2012-06-18

**Authors:** Stephanie D. Biergans, Julia C. Jones, Nadine Treiber, C. Giovanni Galizia, Paul Szyszka

**Affiliations:** 1 Department of Biology-Neurobiology, University of Konstanz, Konstanz, Germany; 2 Department of Biology-Zoology and Evolutionary Biology, University of Konstanz, Konstanz, Germany; University of Missouri, United States of America

## Abstract

Memory is created by several interlinked processes in the brain, some of which require long-term gene regulation. Epigenetic mechanisms are likely candidates for regulating memory-related genes. Among these, DNA methylation is known to be a long lasting genomic mark and may be involved in the establishment of long-term memory. Here we demonstrate that DNA methyltransferases, which induce and maintain DNA methylation, are involved in a particular aspect of associative long-term memory formation in honeybees, but are not required for short-term memory formation. While long-term memory strength itself was not affected by blocking DNA methyltransferases, odor specificity of the memory (memory discriminatory power) was. Conversely, perceptual discriminatory power was normal. These results suggest that different genetic pathways are involved in mediating the strength and discriminatory power of associative odor memories and provide, to our knowledge, the first indication that DNA methyltransferases are involved in stimulus-specific associative long-term memory formation.

## Introduction

Epigenetic mechanisms, such as DNA methylation, are likely candidates for regulating genes involved in memory formation. Epigenetic marks can persist for a long time and can cause changes in gene expression [1]. Recent studies in mammals [2]–[9] and honeybees [10] show that DNA methyltransferases are involved in long-term memory formation. Most of the studies done in mammals focused on the hippocampus region [2]–[4], [6], [8], although some investigated DNA methylation in the amygdala [5], [9] and cortex [7]. The picture emerging from these studies is quite complex. In the hippocampus and cortex there are dynamic changes in methylation patterns in single genes, e.g. in the memory-related factor *bdnf*, after learning [4], [7], [8]. However, only distinct areas in the promoter and exon regions of single genes could be tested so far.

Time scales of DNA methyltransferase action appear to be diverse in different brain areas or in different conditioning paradigms in mammals. For instance, after auditory fear conditioning, there is an upregulation of DNA methyltransferase 3a in the amygdala, but not DNA methyltransferase 3b 90 minutes after training [9]. Both DNA methyltransferase 3a and 3b were upregulated in the hippocampus using contextual fear conditioning only 30 minutes after training [8]. Similarly, an upregulation in DNA metyhltransferase 3 was found in honeybees mushroom bodies 30 minutes after appetitive olfactory conditioning [10]. In mammals long-term memory performance was impaired by DNA methyltransferase inhibition regardless of the brain area investigated, but short-term memory was not [2], [3], [5], [7].

Is DNA methylation differentially involved in short- versus long-term memory formation in insects similar to observations in mammals? And does DNA methylation affect the strength and discriminatory power of memory differently? To address these questions we investigated the effect of inhibiting DNA methyltransferases on associative olfactory learning and memory formation during classical conditioning in honeybees.

Honeybees (*Apis mellifera*) are a widely used model organism for studying learning and memory formation. Bees easily learn to associate an odorant with a sugar reward during classical conditioning. After conditioning, short-term, as well as long-term memory is formed [11]–[14]. Honeybees and mammals have similar molecular mechanisms underlying memory formation. In honeybees, there are several memory traces. Long-term memory can be formed and persists throughout the lifetime of an individual [12]. A single odorant-sugar pairing is sufficient to induce the formation of short-term memory, which is protein synthesis-independent [15]–[17], three odorant sugar pairings are sufficient to induce the formation of protein synthesis-dependent long-term memory [18], [19]. Importantly, even simple odor reward associative learning induces several processes in the nervous system. There is learning induced neural plasticity in the antennal lobe, which corresponds to the vertebrate olfactory bulb [20]–[25], and in the mushroom body, which is a higher order processing center [26]–[29]. This neural plasticity reflects both stimulus-specific, associative and non-associative forms of learning [20]–[24], [27], [29].

Aside from memory formation [10], DNA methylation is involved in honeybee caste development [30]–[32] and shows a task- and age-related pattern [33]. During adulthood DNA methyltransferases are involved in the active forgetting (extinction) of a learned odor sugar association [10]. DNA methylation is mediated by these highly conserved enzymes, DNA methyltransferases [34]–[40], which are present in vertebrates and invertebrates [41], [42]. Given that honeybees possess the relevant DNA methylation machinery [41], [42] and are a well-studied and established learning and memory model, they provide a useful model to investigate epigenetic mechanisms in memory formation.

DNA methylation is involved in long-term memory formation in general [2]–[10]. However, it remains to be shown which specific aspects of long-term memory formation, as e.g. the discriminatory power (i.e. the stimulus-specific long-term memory), are dependent on DNA methylation. We show that DNA methylation is involved in mediating the discriminatory power of an olfactory associative long-term memory, whereas it appears to be negligible in general and short-term memory formation in our study. These findings extend studies in mammals [2]–[9] and honeybees [10] on the role of DNA methyltransferases in memory formation to more specifically investigate different mechanisms contributing to long-term memory formation and to different learning paradigms.

## Results

### DNA methyltransferase inhibition affects the discriminatory power of olfactory long-term, but not short-term memory retrieval

**Figure 1:**
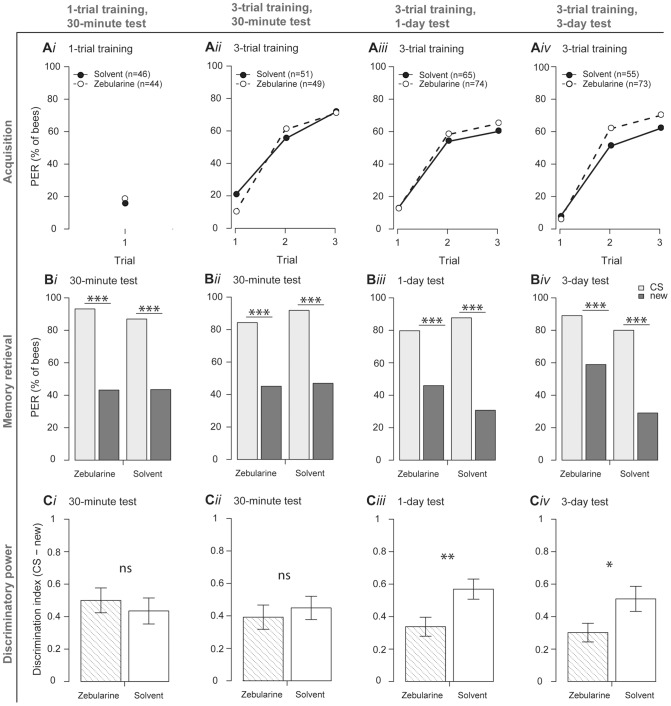
DNA methyltransferase inhibition reduces the discriminatory power of olfactory long-term memory retrieval. Four groups of bees (columns) were trained using appetitive olfactory conditioning and evaluated for acquisition (first row), memory retrieval (second row) and discriminatory power (third row). Each group was divided into two subgroups, one was treated with zebularine and the other was treated with the solvent DMF. Learning was quantified through observations of the conditioned-stimulus (CS) evoked proboscis extension response (PER). The sequence of CS and new odorant was balanced. Discriminatory power of the memory retrieval was quantified as the difference between a bees' response to CS and new odorant (1, PER to the CS only; 0, PER to CS and new, -1, PER to new only). (**A** ***ii-iv***) Zebularine- and solvent-treated bees learned equally well during 3-trial training (p>0.18, Fisher's exact test). In the memory retrieval test (**B** ***i***) 30 minutes after 1-trial training and (**B** ***ii***) 30 minutes, (**B** ***iii***) 1 day and (**B** ***iv***) 3 days after 3-trial training bees responded more to the CS than to the new odorant (p<0.001, McNemar test). Memory retrieval did not differ in discriminatory power between zebularine- and solvent-treated bees when tested 30 minutes after (**C** ***i***) 1-trial training (zebularine group: n = 44; solvent group: n = 46; p = 0.56, Welch's two sample t-test) or (**C** ***ii***) 3-trial training (zebularine group: n = 49; solvent group: n = 51; p = 0.58, Welch's two sample t-test). Memory retrieval was less odor specific in zebularine-treated bees than in solvent-treated bees when tested (**C** ***iii***) 1 day (zebularine group: n = 74; solvent group: n = 65; p = 0.008, Welch's two sample t-test) or (**C iv**) 3 days (zebularine group: n = 73; solvent group: n = 55; p = 0.03, Welch's two sample t-test) after 3-trial training.

To investigate the effect of DNA methyltransferase inhibition on long- and short-term memory formation in honeybees, we conditioned individuals to associate an odorant (conditioned stimulus, CS) with a sucrose reward (Fig. 1a). Memory retrieval was assessed by using the proboscis extension response (PER) [11] to the CS and a new odorant at different time points after training (30 minutes, 1 day, 3 days) (Fig. 1B). These time points were chosen to assess protein synthesis-independent short-term memory (30-minute test, Fig. 1B *i-ii*) and protein synthesis-dependent long-term memory (1- and 3-day test, Fig. 1B *iii-iv*) [12]. Bees were treated with either the DNA methyltransferase inhibitor zebularine dissolved in dimethylformamide (DMF) [43]–[49], or with DMF alone. We found that DNA methyltransferase inhibition did not affect learning during the conditioning procedure (Fig. 1A) or memory strength during the retrieval test (Fig. 1B) in any of the groups. Thus, memory strength does not seem to rely on DNA methylation.

**Figure 2:**
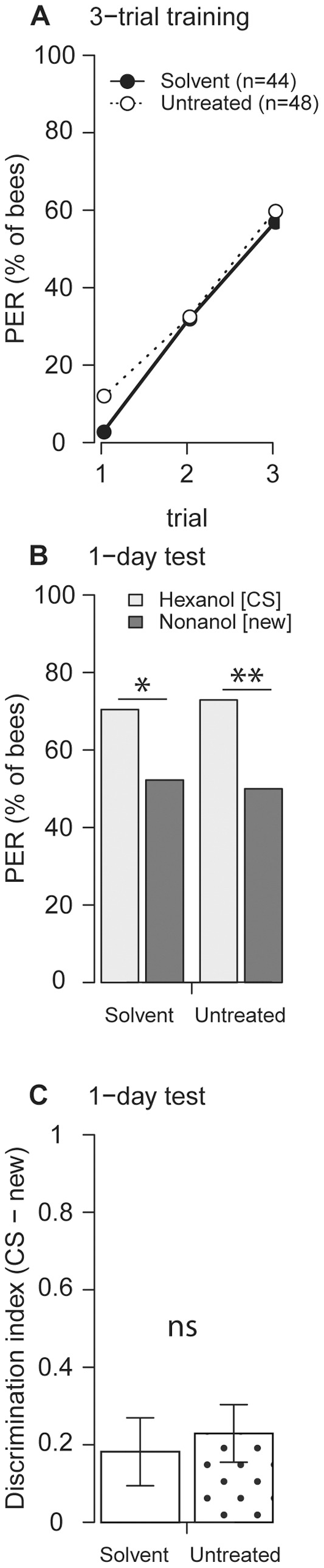
DMF alone does not affect learning and memory retrieval one day after training. (**A**) DMF-treated and untreated bees showed no significant difference in learning during 3-trial training (p>0.1, Fisher's exact test). n indicates the number of bees. (**B**) In the memory retrieval test, 1 day after training, bees responded more to the CS (1-hexanol) than to the new odorant (1-nonanol) (solvent group: n = 44; untreated group: n = 48; p = 0.038 and p = 0.004, McNemar test). The sequence of CS and new odor was balanced. (**C**) Memory retrieval did not differ in discriminatory power between DMF treated and untreated bees (p = 0.68, Welch's two sample t-test).

Next, we quantified the discriminatory power of the memory retrieval by measuring the difference between the response to the CS and a new odorant (Discrimination index, Fig. 1C). Zebularine treatment did not reduce the discriminatory power of the 30-minute memory after 1-trial or 3-trial training (Fig. 1C *i-ii*). In contrast, during the 1- and 3-day memory retrieval test the memory discriminatory power was significantly larger in the solvent-treated group than in the zebularine group (Fig. 1C *iii-iv*). In order to test for possible effects of the solvent DMF on long-term memory formation, we treated bees with DMF or left them untreated and tested their memory retrieval one day after training. DMF treatment alone did not affect learning and memory formation one day after training (Fig. 2).

These results suggest that DNA methylation is required for mediating the olfactory discriminatory power of protein synthesis-dependent long-term memory, but not for general memory strength, or for protein synthesis-independent short-term memory.

### DNA methyltransferase inhibition does not affect perceptual discriminatory power

**Figure 3:**
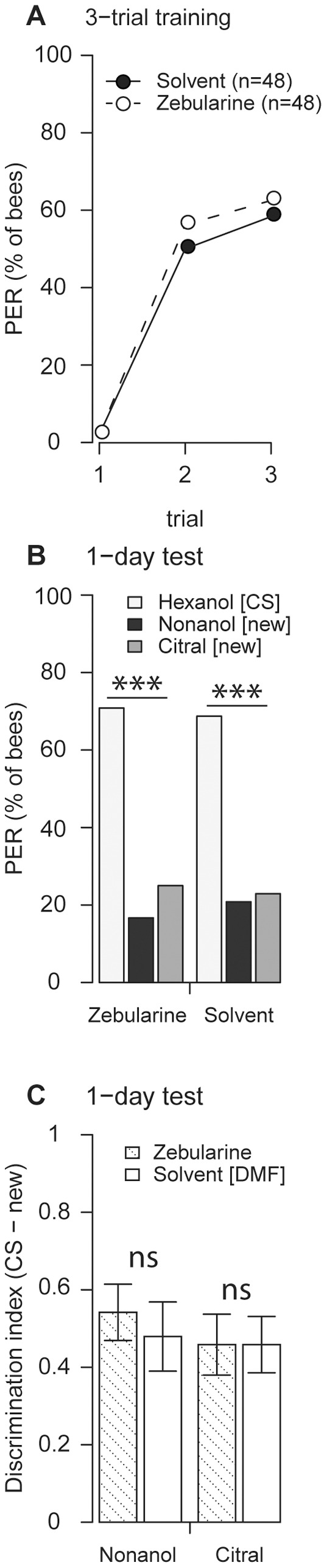
Zebularine treatment does not reduce the perceptual discriminatory power. Bees were treated with zebularine or with the solvent DMF 24 hours before conditioning. (**A**) There was no significant difference in learning between zebularine- and solvent-treated bees during 3-trial training (p>0.68, Fisher's exact test). n indicates number of bees. (**B**) In the memory retrieval test, 1 day after training, bees responded more to the CS (1-hexanol) than to the new odorants 1-nonanol or citral (zebularine group: n = 48; solvent group: n = 48; p<0.001, McNemar test). The sequence of CS and 1-nonanol was balanced; citral was always presented as the last odor. (**C**) Memory retrieval did not differ in discriminatory power between zebularine- and solvent-treated bees (1-nonanol p = 0.59; citral p = 1, Welch's t-test).

DNA methyltransferase inhibition might reduce the olfactory discriminatory power independent of memory formation, which would also lead to a reduced discrimination index in a learning experiment. We therefore tested memory-independent olfactory discriminatory power by treating bees with zebularine 1 day before training and tested their 1-day memory retrieval (Fig. 3). If zebularine treatment reduces the discriminatory power of a bee's odor perception, zebularine-treated bees would retrieve a less odor-specific memory and would generalize more to a new odorant than solvent-treated bees. However, zebularine- and solvent-treated bees did not differ in CS-memory strength (Fig. 3B) or in the discriminatory power (Fig. 3C). Therefore, bees' olfactory perceptual discriminatory power does not appear to be influenced by DNA methyltransferase inhibition.

### Zebularine treatment does not affect bees̀ survival

**Figure 4:**
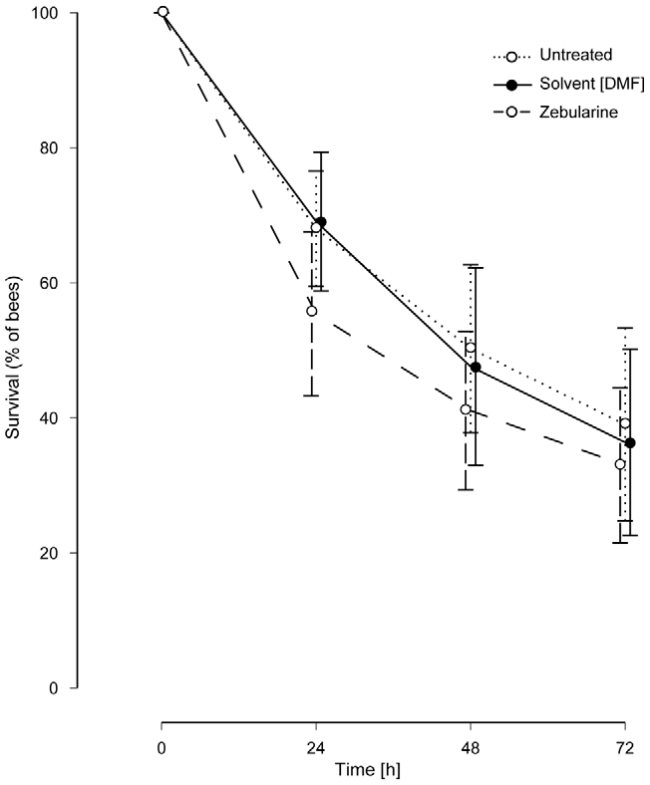
Zebularine treatment does not reduce bee survival. Bees were either treated with zebularine, DMF or were left untreated, and were kept for 72 hours. Every 24 hours the number of living bees was counted. A total of 49 bees were tested per group. The experiment was repeated seven times with 4–10 bees per replicate over a period of six months. The survival rates did not differ between the 3 groups (generalized linear mixed model, factor time: p<0.001, factor DMF treatment: p = 0.887, factor zebularine treatment: p = 0.253).

We controlled for noxious effects of zebularine treatment by comparing the survival of zebularine-, solvent-treated and untreated bees. Zebularine did not significantly affect the survival of the bees up to 3 days after treatment (Fig. 4). Similarly, the solvent DMF did not reduce survival rates. Given the latter results, and that zebularine or DMF treatment did not affect the general ability of bees to respond to the stimuli or being trained (Fig. 1A *i-iv*, 3A), we assume that zebularine treatment does not have a noxious effect on the bees.

## Discussion

We show that the formation of the odor-specific component of protein synthesis-dependent olfactory long-term memory in honeybees involves DNA methylation-dependent gene regulation. Long-term memory strength and protein synthesis-independent short-term memory formation were not impaired by DNA methyltransferase inhibition in our study. We believe this is the first demonstration of the involvement of an epigenetic mechanism in stimulus-specific associative memory formation and the first demonstration that DNA methyltransferases are not involved in short-term memory formation in honeybees. The latter result is in accordance with studies conducted in mammals, where it has been suggested that DNA methylation may play a role in long-term, but not short-term memory formation [2], [3], [5], [7]. DNA methyltransferase inhibition did not change odor perception *per se* as the perceptual discriminatory power was unchanged (Fig. 3). There also was no effect on survival rates up to three days after treatment (Fig. 4), which would indicate a noxious effect of zebularine treatment.

The reduced discriminatory power of the bees' retrieved long-term memory after zebularine treatment likely reflects the formation of a less odor-specific memory. Because odor-specific memory formation alone was impaired by DNA methyltransferase inhibition (Fig. 1), we assume that there may be different genetic pathways involved in mediating discriminatory power compared with the associative but odor-unspecific strength of an odor memory. Thus, the gene pathway, which mediates memory-discriminatory power, seems to be at least partly regulated by DNA methylation.

We did not detect any effect of DNA methyltransferase inhibition on a bee's ability to learn an odor (memory strength, Fig. 1B). This may indicate that DNA methyltransferases are not necessary for being able to associate a sugar-reward with an odor in general. However, Zebularine only partly inhibits DNA methyltransferase activity and we only used one concentration of the inhibitor, inhibited at a specific time point and used a non-invasive application method. Thus it is possible that DNA methylation is important for memory strength as well, but we did not detect an effect here. Discriminatory power may be one of the properties of long-term memory that is affected even with a weak inhibition of DNA methyltransferases. It may need much stronger inhibition of DNA methylation to impair memory strength. Another possibility is that memory strength and discriminatory power rely on DNA methylation at different time points and that we therefore did not detect any effect on memory strength, as we tested only one inhibition time point (Fig. 1A). Further studies are required to address this.

By comparison, in mammals DNA methyltransferase inhibition impairs the general ability to learn during contextual fear conditioning, where a context is associated with an electric shock [2], [4], [6]–[8]. This shows that DNA methyltransferases are involved in memory formation after aversive conditioning [2], [4], [6]–[8]. Our study together with others shows that DNA methyltransferases are involved in appetitive learning [10] as well, whereas the dynamics of DNA methyltransferase regulation are likely to be different in different learning paradigms [8]–[10].

In the honeybee, only the discriminatory power of long-term memory was impaired after DNA methyltransferase inhibition (Fig. 1) suggesting that DNA methyltransferases are only involved in some, but not all mechanisms contributing to the formation of long-term memory in the same way. DNA methylation-dependent gene regulation may take place at different time points during memory acquisition and consolidation. The time point of DNA methyltransferase inhibition in relation to the training period may be relevant for our findings. The effect of different time points of inhibition on extinction retention has been shown previously [10]. Inhibition time is also likely to influence the effect of DNA methyltransferase inhibition on associative long-term memory retention, as there are other processes that can influence DNA methylation besides learning [30]–[32]. It will therefore be interesting to narrow down the time point at which DNA methylation-dependent gene regulation of long-term memory formation takes place.

We note that DNA methyltransferases have previously been shown to be involved in associative memory processing in honeybees [10]. In that study, zebularine treatment impairs long-term odor memory strength and extinction retention in young bees (7 days). The difference between the latter results and those of the current study may reflect the difference in learning paradigms and experimental protocols used, since that study used a differential conditioning protocol followed by extinction trials to assess both memory retention after one day and extinction retention after inhibition of DNA methyltransferases at different timepoints. In addition, the age of the bees differed between the studies, where bees in the current study were foragers and therefore older and more experienced. As the bees used in our study were not age matched the different individuals may have had different preexisting olfactory experiences. This may cause variation in responsiveness, learning and memory performances, which could hide possible effects of DNA methyltransferase inhibition. To exclude systematic effects of age difference, we conditioned all experimental groups in parallel.

Another interesting connection investigated recently is the association between DNA methylation and the Proteinkinase C system, which is involved in synaptic plasticity in mammals [3] and is also involved in long-term memory formation in honeybees [15]. Further analysis investigating, how these systems are linked and which genes are regulated by DNA methylation during long-term memory formation will be an interesting challenge, for which the honeybee seems to be a suitable model organism. Because only the discriminatory power of bees was affected by DNA methyltransferase inhibition, it will be interesting to conduct a genome-wide search for differentially methylated genes involved in this specific memory trace. The fact that different mechanisms of long-term memory formation are differently influenced by DNA methylation in the honeybee may be highly relevant for uncovering the role of DNA methylation in mammals. To date, all studies on DNA methylation and memory formation in mammals have focused on the effect of DNA methylation on memory strength [2]–[9]. Discriminatory power has not been tested. Inhibition of DNA methyltransferases does not prevent memory formation entirely, rather it does so to varying degrees [2], [4]–[10]. Thus, it is likely that certain features of memory formation are DNA methyltransferase-independent or less dependent and others are highly dependent both in mammals and honeybees.

### Conclusion

We investigated the role of DNA methylation in learning and memory formation in honeybees. We found that DNA methyltransferases are likely to be involved in long-term memory processing in honeybees, but not in short-term memory formation. Treatment with zebularine affected the discriminatory power of bees' long-term memory but did not affect memory strength suggesting that different genetic pathways are involved in odor-specific and general memory formation. Long-term memory is increasingly appearing to be a complex interplay between different brain areas, cell populations, and – as shown here – epigenetic mechanisms.

## Methods

### Conditioning procedure

Free flying honeybees (*Apis mellifera*) were caught inside a bee house from one single hive. For a detailed description of bee handling, conditioning procedure and odorant stimulation see Szyszka et al. [50]. Classical conditioning was performed with either 1-hexanol or 1-nonanol as conditioned odor (CS) (Fig. 1) or only 1-hexanol as CS (Fig. 2,3). The odorants were diluted 10^−2^ in mineral oil (Sigma-Aldrich, Deisenhofen, Germany). 100 µl of the odorant solution was applied to a 1cm^2^ piece of cellulose (Sugi pads, Kettenbach, Eschenburg, Germany) and placed in the olfactometer. Four-second long odorant stimuli were given with a custom-made olfactometer. The CS was paired with 1 M sucrose solution as a reward, which was presented 2 seconds after CS onset for 3 seconds. The inter-trial interval was 10 minutes. Bees either experienced one-trial conditioning or three-trial conditioning. Memory retrieval was tested 30 minutes, 1 day or 3 days after training. Every bee was tested only once. During each test, the CS and a new odorant were presented. The sequence of CS and new odorant was balanced in order to exclude sequence effects. 1-hexanol and 1-nonanol were used equally as CS and new odorant (Fig. 1). The different experimental groups were always conditioned in parallel to exclude differences due to daily variation in performance.

### Pharmacological treatment

Harnessed bees were treated with either the DNA methyltransferase inhibitor zebularine (Tocris, Ellisville, USA) dissolved in DMF with a concentration of 2 mM or the carrier DMF. Zebularine is an inhibitor for DNA methyltransferases [43]–[49], which is most potent for DNA methyltransferase 1, but also affects DNA methyltransferase 3 [44], [46]. It increases the binding affinity of DNA methyltransferases to the DNA so that they are not active anymore [44]. Bees were treated 1 hour before and immediately after training (Fig. 1, 2). During the test for the effect of zebularine on perception bees were treated twice with 1 µl zebularine or DMF with a 90-minute gap between applications. Bees were trained 1 day after the treatment (Fig. 3). During the survival test bees were treated twice with 1 µl of zebularine or DMF or they were left untreated with a 90 minute gap between treatments. The 1 µl drop of the drug was applied topically on the back of the thorax with a pipette, as done in a previous study [10].

### Survival test

Bees were treated with 1 µl zebularine or DMF twice with a 90 minute gap between treatments. Bees were treated in line with the handling during the conditioning experiments. A third group was left untreated. Bees were kept in a humid plastic box for three days and were fed *ad libitum* every evening. 24, 48 and 72 hours after treatment, the number of surviving bees was counted. The survival of all three groups was measured in parallel.

### Data analysis

Data were analyzed with custom-written programs in R (R.2.13.1, The R Development Core Team, 2011).

For the comparisons between the responses to a specific odor of zebularine- and solvent-treated bees, the Fisher's exact test was used. This test is appropriate for unpaired binary data. For comparisons between the conditioned and the new odorant within a treatment group the McNemar test was used. The latter test is appropriate for the comparison of paired binary data. The discrimination indexes of the different treatment groups were compared using Welch's t-test. The discrimination index was calculated by subtracting the response of a single bee to the new odor from the response to the CS. Therefore a value of -1 equals a response only to the new odor, 0 to both odors and 1 only to the CS. The survival test was analyzed using a generalized linear mixed model with binomial error distribution and logit-link function to analyze the proportion of bees that have survived at three time points for each treatment group. Treatment, time and trial number were used as predictor variables. Interactions between factors, trends with the progress of the season, and overdispersion were not present. All data analysis for the survival test was done in R 2.15.0.
